# Clinical efficacy maintains patients’ positive attitudes toward fecal microbiota transplantation

**DOI:** 10.1097/MD.0000000000004055

**Published:** 2016-07-29

**Authors:** Lijuan Xu, Ting Zhang, Bota Cui, Zhi He, Jie Xiang, Chuyan Long, Zhaoyuan Peng, Pan Li, Guangming Huang, Guozhong Ji, Faming Zhang

**Affiliations:** a Medical Center for Digestive Diseases, the Second Affiliated Hospital of Nanjing Medical University; b Key Laboratory of Holistic Integrative Enterology, Nanjing Medical University, Jiangjiayuan, Nanjing, Jiangsu Province, China.

**Keywords:** attitude, Crohn's disease, fecal microbiota transplantation, questionnaire

## Abstract

Few studies have been conducted on the attitudes of patients seeking fecal microbiota transplantation (FMT). This study aimed to investigate the reasons for patients with Crohn's disease (CD) seeking FMT and their attitude changes after FMT.

In this prospective study, all included patients were diagnosed with CD for at least 6 months and intended to receive FMT. A questionnaire was designed to investigate the history of medical visits and patients’ attitudes toward FMT. Only refractory patients who failed to clinically respond to previous treatment were selected for undergoing FMT. Three months after the first FMT, patients were required to complete the second questionnaire on attitudes toward the first FMT.

A total of 207 patients with CD were included for questionnaire survey. In 118 refractory patients, 94.07% sought FMT because they had no other choice. In 89 nonrefractory patients, 78.65% sought FMT for the reason that they wanted to achieve better clinical results or even a cure, although the current treatment was effective for them. In all, 118 refractory patients received FMT. Three months after the first FMT, 88.98% (105/118) patients completed the questionnaire on patients’ attitudes toward FMT. Of these 105 patients, 56.19% reported to have satisfactory clinical efficacy and 74.29% were willing to receive the second FMT. Moreover, 89.52% (94/105) showed their willingness to recommend FMT to other patients.

In conclusion, this study at least first time demonstrated that patients with CD were willing to accept FMT due to its efficacy.

## 1 Introduction

Crohn's disease (CD), a phenotype of inflammatory bowel disease (IBD), is a chronic relapsing and remitting inflammatory disorder of the gastrointestinal tract with unknown etiology that affects mainly young adults. The incidence and prevalence of CD have been reported to be greatly increasing in many regions of Asia since the 1990.^[1–3]^ However, there is no cure for CD at present, and a number of studies are exploring new treatment strategies.

Though the pathogenesis of IBD is currently unknown, it is generally accepted that dysbiosis of gut microbiota may play a pivotal role in the development of chronic inflammation in IBD.^[4]^ Fecal microbiota transplantation (FMT) is a novel treatment that has raised increasing attention in recent years. FMT, involving the infusion of fecal suspension from a healthy individual into patients’ intestine, has been reported as a potential therapy for IBD.^[5,6]^ The aim underlying FMT is to restore gut microbiota. The efficacy of FMT in CD is still controversial.^[7–10]^ Vermeire et al^[10]^ reported that 4 patients with CD did not experience clinical improvement after FMT. However, our previous study on FMT for refractory CD showed a high rate of clinical improvement (80%) and remission (70%) at the third month.^[11]^

Several studies have reported that patients were willing to consider FMT as an alternative treatment for recurrent *Clostridium difficile* infection and IBD.^[12,13]^ However, there was no study targeting attitude changes toward FMT in patients with IBD. Based on our clinical trial (NCT01793831) for CD, this study aimed to investigate the reasons for patients with CD seeking FMT and their attitude changes after FMT.

## 2 Materials and methods

### 2.1 Patients and methods

This study was performed at the Second Affiliated Hospital of Nanjing Medical University from November 2012 to September 2015. The study protocol was approved by the institutional ethical committee. All included patients were diagnosed as having CD and assessed using Harvey–Bradshaw Index (HBI) score.^[14]^ Clinical classification of CD was performed according to the Montreal classification.^[15]^ Disease location was classified as ileal (L1), colonic (L2), ileocolonic (L3), or isolated upper gastrointestinal (L4) disease. Disease behavior was classified as nonstricturing and nonpenetrating (B1), stricturing (B2), and penetrating (B3) despite perianal involvement (P).

**Figure 1:**
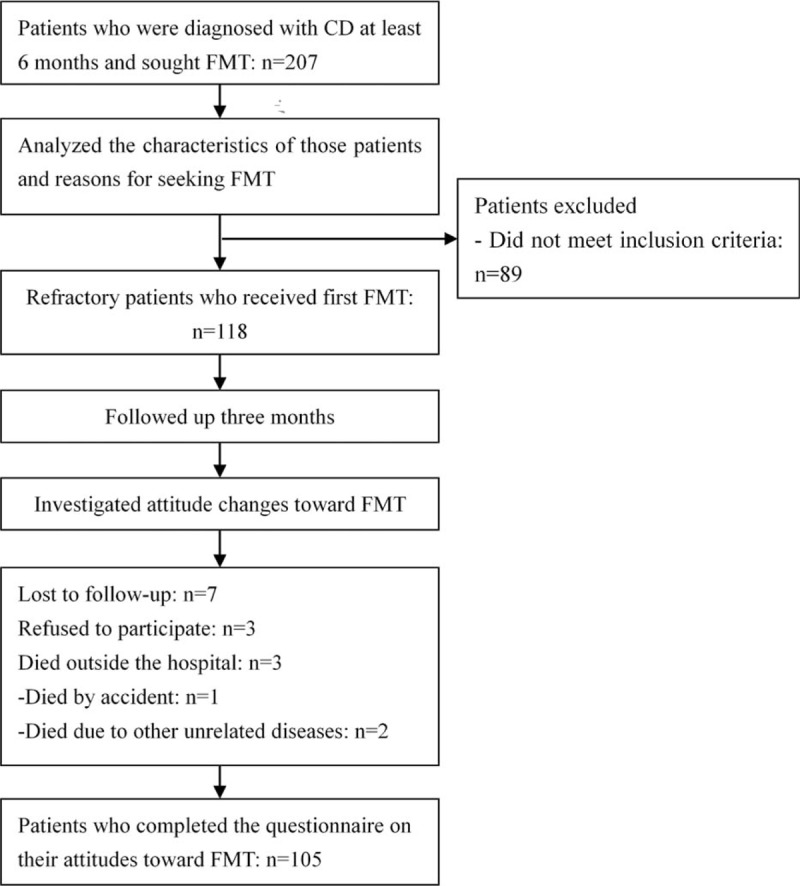
Flow chart of the study.

All included patients aimed to seek FMT and had been diagnosed with CD at least 6 months. The medical records, endoscopic, radiological, and histological examinations were reviewed for collecting data. A questionnaire was used to investigate the history of treatment and the reasons for seeking FMT in all patients. The following information was extracted from all data source above: baseline characteristics; history of treatment; reasons for seeking FMT; attitude changes after the first FMT. However, only refractory patients were accepted to undergo FMT. The inclusion and exclusion criteria for refractory CD^[11]^ were shown in Table 1. A questionnaire was required to investigate their attitude changes 3 months after the first FMT. Flow chart of the study was shown in Fig. 1.

**Table 1:**
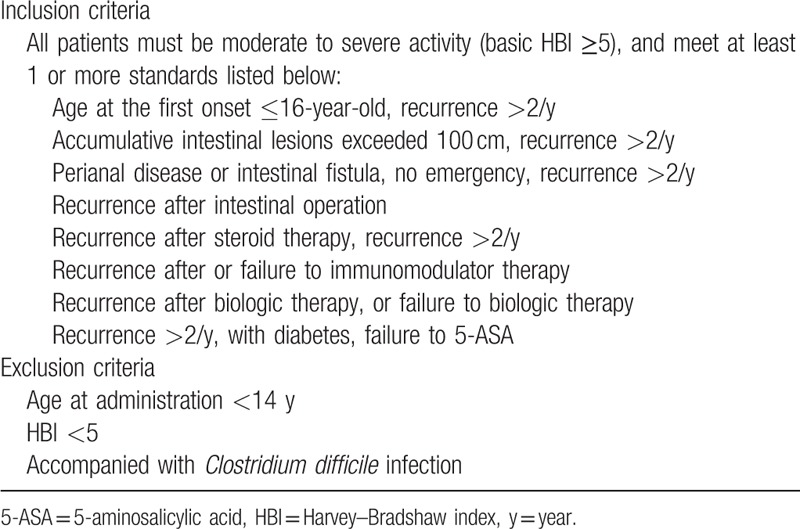
Inclusion criteria and exclusion criteria for refractory Crohn's disease.

### 2.2 Statistical analysis

Statistical analysis was performed using SPSS version 15.0. Continuous variables were expressed using mean ± standard deviation (SD) or median. Categorical variables were summarized using absolute numbers and percentages.

## 3 Results

### 3.1 Demographic data

**Figure 2:**
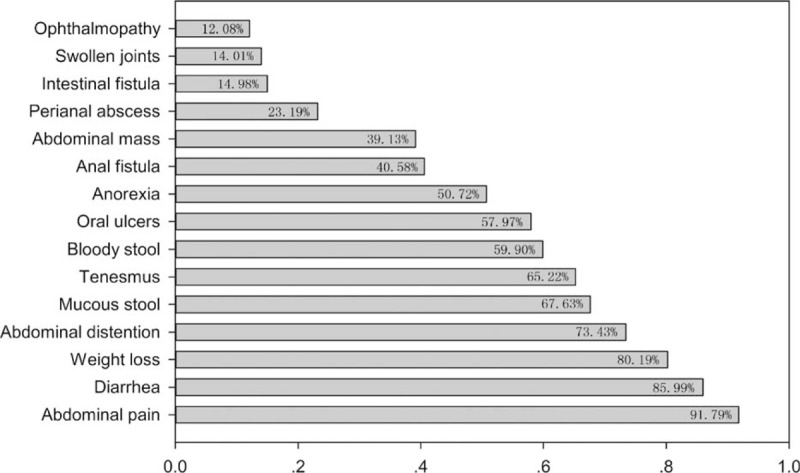
The profiling of Crohn's disease-related clinical symptom or diseases (n = 207).

In total, 207 patients with CD (132 males and 75 females) were investigated in this study. The basic characteristics of all enrolled patients were summarized in Table 2. The mean age was 33.94 ± 14.50 years. Among the patients, 31.88% (66/207) had moderate and 43.48% (90/207) had severe CD. Moreover, 48.79% (101/207) had ileocolonic involvement. Stricturing and penetrating behavior were reported in 95 patients (45.89%) and 31 patients (14.98%), respectively. Perianal lesions were confirmed in 89 patients (43.0%). The profiling of CD-related clinical symptom or diseases is shown in Fig. 2. The most common symptom was abdominal pain (91.79%), followed by diarrhea (85.99%) and weight loss (80.19%). In addition, 14.98% (31/207) had intestinal fistula and 73.43% (152/207) had extraintestinal symptoms.

**Table 2:**
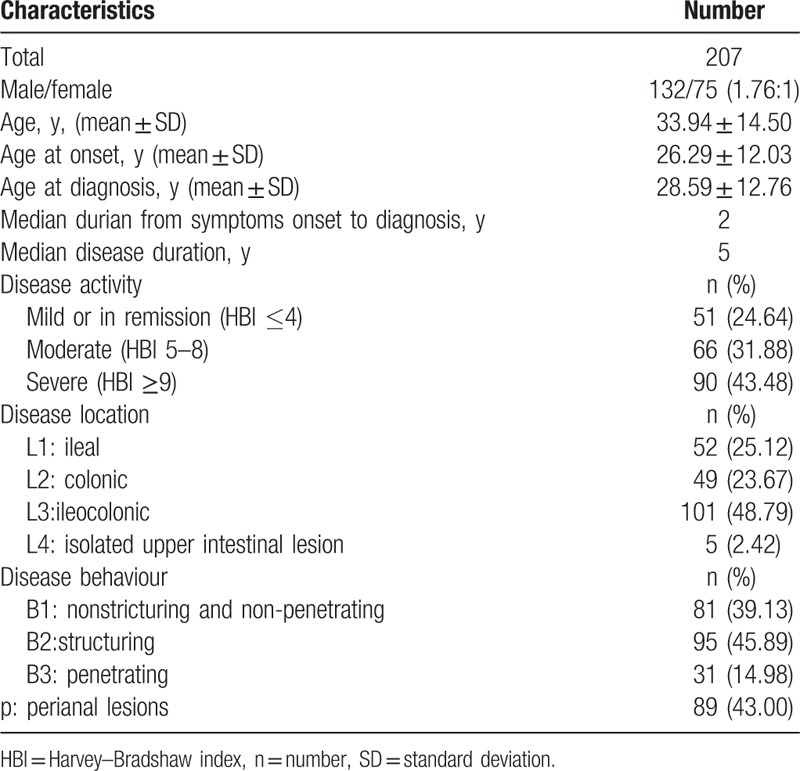
Characteristics of all 207 patients with Crohn's disease seeking fecal microbiota transplantation.

### 3.2 History of treatment

**Figure 3:**
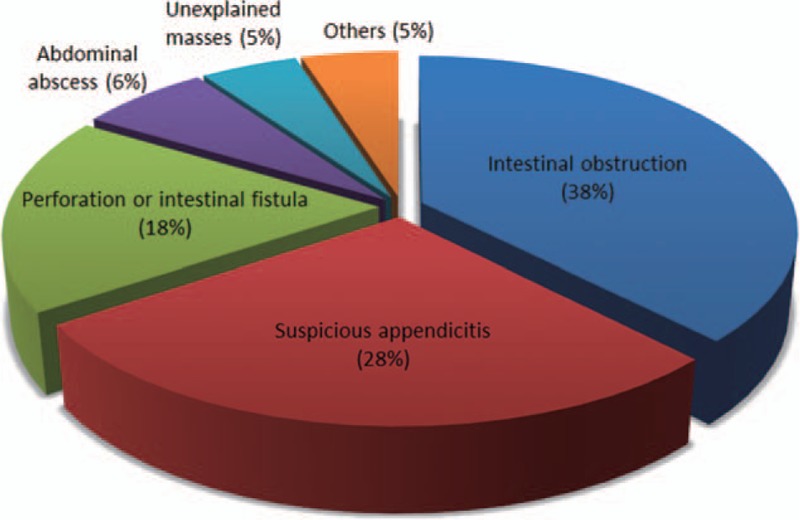
Reasons for intestinal surgery (not including perianal surgeries or hemorrhoidectomy) in patients with Crohn's disease (n = 52).

Based on the selected population of this study, the median number of hospitals patients had visited was 6 (range 2 to 14). In total, 25.12% (52/207) underwent at least 1 intestinal surgery (not including perianal surgeries or hemorrhoidectomy). Reasons for intestinal surgeries were shown in Fig. 3.

The medications that patients had received from establishment of the diagnosis to ahead of our center are listed in Table 3. Among the patients, 87.44% (181/207) took mesalazine. 57.00% (118/207) took corticosteroids, and 40.58% (84/207) took thiopurine. Only 15.94% (33/207) used infliximab.

**Table 3:**
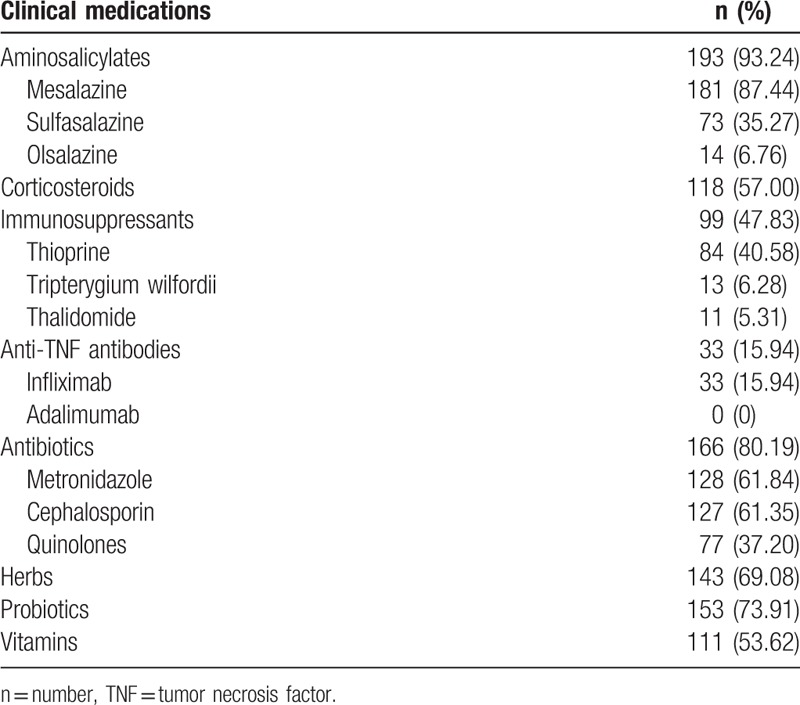
Clinical medications in patients with Crohn's disease (n = 207).

### 3.3 Reasons for seeking FMT

All patients coming to our center wanted to accept FMT. In 118 refractory patients, 94.07% (111/118) sought FMT because they had no other choice (Table 4). In 89 nonrefractory patients, 78.65% (70/89) sought FMT for the reason that they wanted to achieve better clinical results or even cure, although their current treatment was effective for them.

**Table 4:**
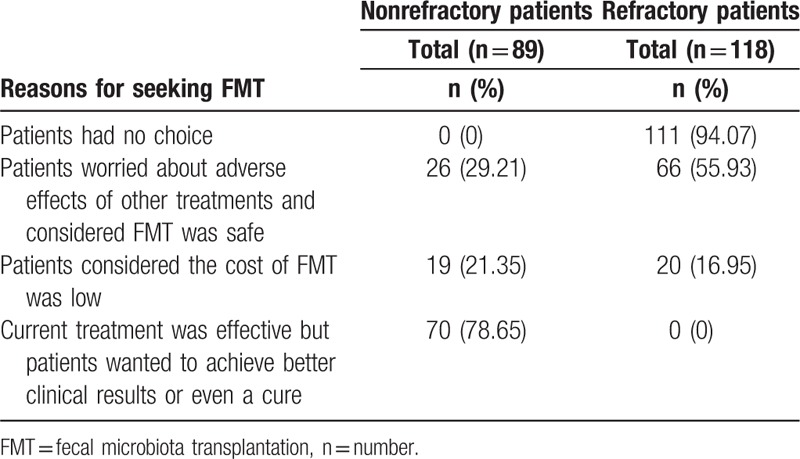
Reasons for seeking fecal microbiota transplantation.

### 3.4 Attitude changes after the first FMT

A total of 118 refractory patients underwent FMT. Three months after the first FMT, 7 patients were lost to follow-up, 3 refused to complete the questionnaire, and 3 died outside of the hospital (1 died by accident and 2 died of other unrelated diseases) (Fig. 1). Finally, 105 of 118 patients (88.98%) completed the questionnaires on patients’ attitudes toward the first FMT (Fig. 1). Of these 105 patients, 78 (74.29%) were willing to undergo the second FMT and 59 (56.19%) reported to have satisfactory clinical results (Table 5); 17.14% (18/105) who did not show a satisfactory response to the first FMT were still willing to undergo the second FMT. On the contrary, 25.71% (27/105) were unwilling to undergo the second FMT, because they achieved transient (8.57%) or even no (17.14%) clinical efficacy, and 89.52% (94/105) showed their willingness to recommend FMT to other patients.

**Table 5:**
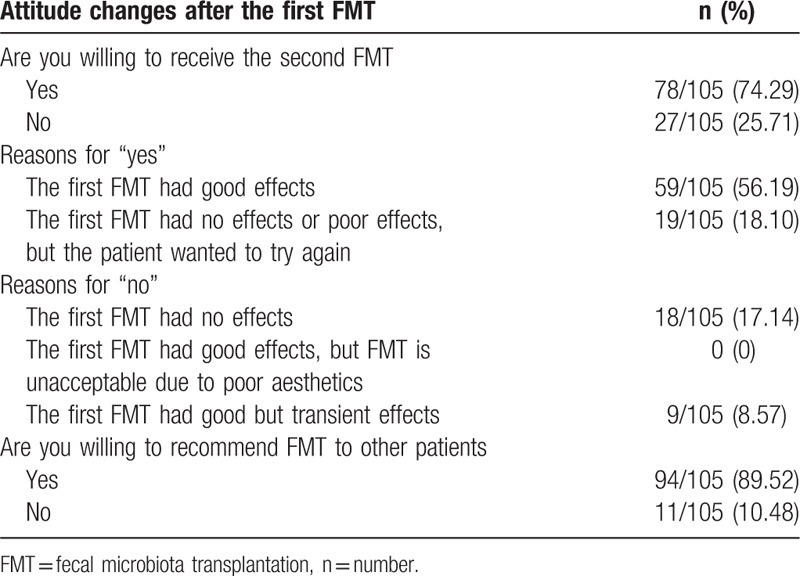
Attitude changes after the first fecal microbiota transplantation.

## 4 Discussion

In this study, the history of treatment and characteristics of the included patients showed their complex situation. The male-to-female ratio and the operation rate were similar to the rates from the Asia Pacific Consensus Statements.^[16]^ However, the rate of extraintestinal symptoms (73.43%) in this surveyed population was higher than the common rate (approximately 20%–40%).^[16]^ Besides, this study displayed a high usage rate of herbs. The concept of FMT has been described in traditional Chinese medicine at least since the fourth century,^[17]^ which may be one of the reasons why patients believed and sought FMT.

This study investigated the reasons for patients with CD seeking FMT. Among the refractory patients, 94.07% (111/118) wanted to undergo FMT because they had no other choice, and 55.93% (66/118) wanted to undergo FMT because they worried about adverse effects of other treatments and thought FMT was safe. Nowadays, traditional treatments for CD included aminosalicylates, corticosteroids, immunosuppressants, biological agents (mainly infliximab in China), antibiotics, and surgeries. Corticosteroids are generally prescribed for refractory patients and have been proven highly effective in acute attacks of CD.^[18,19]^ Unfortunately, steroids have many serious adverse effects (e.g., osteonecrosis, osteoporosis, severe infections, and psychiatric complications).^[20–23]^ Steroid-dependence in patients with CD is also an important clinical problem. Infliximab is one choice for refractory luminal and fistulizing CD, and also extraintestinal manifestations,^[24]^ but its use is restricted due to the high incidence of viral hepatitis and tuberculosis,^[24–26]^ and the high price without medical insurance coverage in previous years. Surgical interventions would be needed for patients with CD accompanying serious complications. In our trial, FMT was not considered if patients need surgical intervention. It was previously hypothesized that patients who were taking medications (such as mesalazine) for maintaining remission without serious adverse effects or who were mild or in remission would not be interested in FMT. However, the findings from the present study showed that FMT was still an attractive option for them. We proposed that FMT might be a promising rescue therapy for refractory CD.^[27]^ Therefore, our registered clinical trials claimed to recruit patients with refractory CD.^[11]^

This study also showed the positive attitudes toward FMT in patients with CD. Three months after the first FMT, 74.29% (78/105) of patients who had received the first FMT were willing to receive the second FMT and 56.19% (59/105) reported to have satisfactory clinical results. Most importantly, we found that FMT showed a fast and continuous significant effect in relieving the sustaining abdominal pain associated with sustaining CD.^[11]^ This should be helpful to improve patients’ life quality. Nineteen of the 105 patients (18.10%) who did not show a satisfactory response to the first FMT were still willing to receive the second FMT. They believed that 1 FMT may not be enough. But 25.71% of patients who had received the first FMT were unwilling to receive the second FMT. Eighteen of the 105 patients (17.14%) said “no” to the second FMT because they did not achieve clinical efficacy or temporary clinical improvement. It was reported FMT is “somewhat unappealing” to some patients owing to its nature.^[12]^ On the contrary, none refused to accept the second FMT for the reason of poor aesthetics in this study. In our center, we performed FMT through mid-gut or colonic transendoscopic enteral tubing (TET),^[28]^ which may improve the understanding of FMT and eliminate patients’ concerns about the aesthetics of FMT. It was expected that patients who were unwilling to receive the second FMT would also refuse recommending FMT to other patients. However, this study showed 89.90% (94/105) were still willing to recommend FMT to others. During our survey, we found that this reason is possibly related to patients’ personal judgment that FMT may not be suitable for themselves, but would be worth recommending to others.

There are several limitations in this study. This is a single-center study with limited number of cases. Larger sample is necessary for future researches. Furthermore, the questionnaire on FMT is not investigated by a third party during follow-up. Although we had emphasized to patients that this survey was just used for research, the report from few patients may have bias. Some of these investigated patients underwent FMT through TET technique, because the survey results may change since they experienced TET as colonic delivering way of FMT.^[28]^ Additionally, this study only indicated the current attitudes of patients on FMT. With the improvement of human understanding on FMT, the patients’ attitudes may change in the future.

In conclusion, this study investigated the reasons for patients with CD seeking FMT and their attitude changes toward FMT. The results at least first time demonstrated that patients were willing to accept FMT and recommend FMT to other patients due to its efficacy.
